# Endothelial Cell Surface Expressed Chemotaxis and Apoptosis Regulator (ECSCR) Regulates Lipolysis in White Adipocytes via the PTEN/AKT Signaling Pathway

**DOI:** 10.1371/journal.pone.0144185

**Published:** 2015-12-21

**Authors:** Sreenivasulu Kilari, Stephanie Cossette, Shabnam Pooya, Michelle Bordas, Yi-Wen Huang, Ramani Ramchandran, George A. Wilkinson

**Affiliations:** 1 Department of Pediatrics and Developmental Vascular Biology Program, Medical College of Wisconsin and Children’s Research Institute, Milwaukee, Wisconsin, United States of America; 2 Department of Obstetrics and Gynecology, Medical College of Wisconsin and Children’s Research Institute, Milwaukee, Wisconsin, United States of America; GDC, GERMANY

## Abstract

Elevated plasma triglycerides are associated with increased susceptibility to heart disease and stroke, but the mechanisms behind this relationship are unclear. A clearer understanding of gene products which influence plasma triglycerides might help identify new therapeutic targets for these diseases. The Endothelial Cell Surface expressed Chemotaxis and apoptosis Regulator (ECSCR) was initially studied as an endothelial cell marker, but has recently been identified in white adipocytes, the primary storage cell type for triglycerides. Here we confirm ECSCR expression in white adipocytes and show that *Ecscr* knockout mice show elevated fasting plasma triglycerides. At a cellular level, cultured 3T3-L1 adipocytes silenced for *Ecscr* show a blunted Akt phosphorylation response. Additionally we show that the phosphatase and tensin homology containing (PTEN) lipid phosphatase association with ECSCR is increased by insulin stimulation. These data suggest a scenario by which ECSCR contributes to control of white adipocyte lipolysis. In this scenario, white adipocytes lacking *Ecscr* display elevated PTEN activity, thereby reducing AKT activation and impairing insulin-mediated suppression of lipolysis. Collectively, these results suggest that ECSCR plays a critical function in regulating lipolysis in white adipose tissue.

## Introduction

Adipose tissue serves as the body’s largest energy reservoir, storing fats during energy surplus and releasing fats as energy is needed. In white adipocytes, surplus energy is chiefly stored as triglycerides (TGs) made up of free fatty acids esterified to glycerol. The balance between lipogenesis and lipolysis in adipocytes is regulated by insulin and catecholamines, and by additional hormonal, autocrine and paracrine factors [1]. Catecholaminergic stimulation and insulin-induced inhibition exert opposing effects on the lipolytic activity of hormone sensitive lipase (HSL) [2]. Catecholamine induces HSL phosphorylation, favoring lipolysis, while insulin signaling inhibits HSL phosphorylation and lipolysis via a phosphatidylinositol-3-kinase (PI3K)/v-akt murine thymoma viral oncogene homolog (AKT)-dependent pathway. The PI3K/AKT pathway is further regulated by the lipid phosphatase phosphatase and tensin homology containing (PTEN), which acts to reduce the PI3K stimulation of AKT [3]. Impaired insulin responses in white adipocytes lead to excess circulating lipids, which in turn are associated with inflammatory processes leading to metabolic syndrome [4].

Our lab has been studying Endothelial-Cell Surface expressed Chemotaxis and apoptosis Regulator (ECSCR), which was initially identified as an endothelial marker enriched in both tumor and inflammatory vascular contexts [5–7]. ECSCR expression has since been reported on a variety of normal and transformed cell types [7, 8]. ECSCR encodes a single-pass transmembrane domain cell surface protein lacking homology to other known proteins. At a cellular level, ECSCR operates in tandem with Vascular Endothelial Growth Factor Receptor-2 (VEGFR-2) [8, 9] and other receptor tyrosine kinases [10] to control cellular behaviors. A recent paper by Akakabe et al [11] explored a new role for endothelial ECSCR, namely in the endothelial response to insulin signaling. Those authors reported ECSCR expression in white adipose tissue (WAT), but did not describe differences in white adipocyte function in mice lacking *Ecscr*. Here we show that white adipocytes lacking *Ecscr* are functionally impaired in control of lipolysis. Fasting animals lacking *Ecscr* show increased circulating lipids, both as esterified triglycerides and as free fatty acids. Phosphorylation of HSL is elevated in WAT of these animals, while activating phosphorylation of AKT is reduced. Cultured white adipocytes show enhanced glycerol release and blunted insulin control of lipolysis, while catecholamine responses were not affected. Mechanistically, we show that PTEN association with ECSCR is increased by insulin stimulation and blocked by an inhibitor of PI3K. We propose that ECSCR-PTEN interactions in white adipocytes modulate the AKT pathway downstream of insulin to enhance lipolysis. In this scenario, in white adipocytes lacking *Ecscr*, elevated PTEN activity reduces AKT activation, thereby impairing basal and insulin-dependent suppression of lipolysis. These results suggest a new role for ECSCR within white adipocytes in regulating lipolysis.

## Materials and Methods

### Reagents

Anti–HA antibody was obtained from Covens, anti-PTEN antibody was obtained from Millipore, HRP coupled anti GAPDH antibody was obtained from Santa Cruz Biotechnologies, and anti-β-actin antibody was from Sigma Aldrich. Polyclonal anti-ECSCR antiserum was raised in rabbits as described [8]. All other antisera were obtained from Cell Signaling Technologies unless otherwise specified. 3T3-L1 cells and HEK293T cells were obtained from ATCC. Insulin was obtained from Roche, isoproterenol hydrochloride and other inhibitors were from Sigma-Aldrich. Lipofectamine 2000, DMEM with high glucose, fetal bovine serum, and other cell culture media supplements were from Life Technologies. Recombinant ECSCR cDNA constructs were designed based on human ECSCR (GenBank^TM^accession no. NP_001071161). Sequence details of the ECSCR-ΔC truncation construct were reported previously [8]. PTEN expression construct with C-terminal FLAG tag was purchased from OriGene.

### ECSCR shRNA design and production of lentiviral particles

Targeting sequences were identified (CCACCAGCCTTCAGACAAA and AAGACAGCATCACACTCATCTC), shRNA oligos were synthesized and cloned into pLKO.1 vector, and lentiviral particle production was performed according to Addgene standard protocol (http://www.addgene.org/tools/protocols/plko/).

### Generation and validation of ECSCR knockout mouse

Mouse experiments were approved by The Medical College of Wisconsin IACUC (Animal Protocol AUA1022). ES cells heterozygous for an *Ecscr* “Knockout first” targeting allele (Project number CSD24988; Supplementary Fig 1A) were obtained from the Knockout Mouse Project (KOMP) [12]. The knockout first allele consists of a large cassette containing neomycin resistance and β-galactosidase open reading frames inserted downstream of the conserved exon 2[13]. This large insertion results in a protein null allele (**Panel B** in **S1 Fig**.).

**Fig 1:**
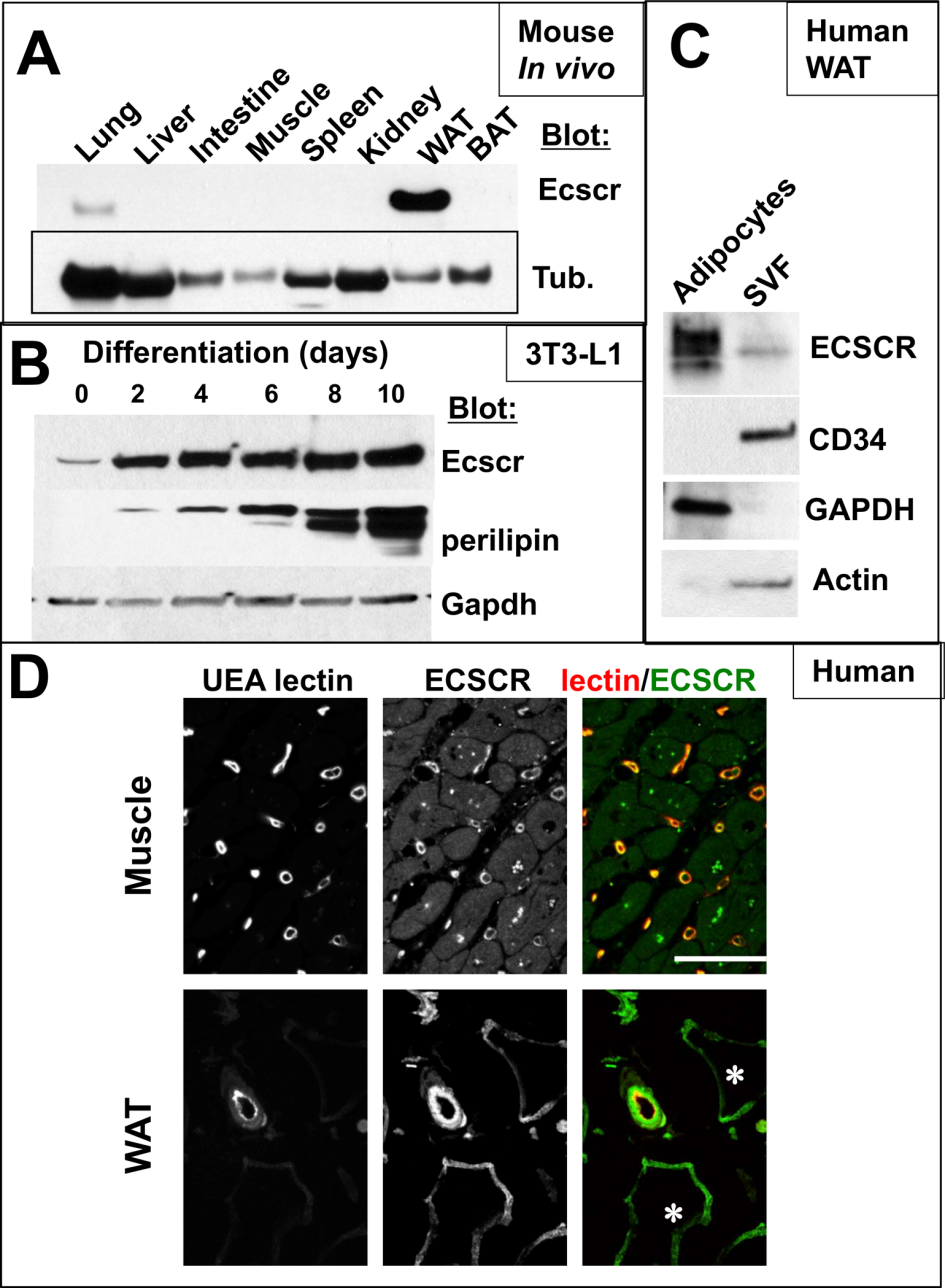
Tissue expression of ECSCR protein in mouse and human white adipocytes. **A.** Anti-ECSCR western blot of adult male mouse tissues. Results are representative of lysates from three independent animals. WAT, epididymal white adipose tissue. Tub, tubulin loading control. **B.** ECSCR protein in cultured 3T3-L1 cells during differentiation to adipocytes. ECSCR protein is compared to reference lipid droplet marker perilipin, with GAPDH as loading control. **C.** Fractionation of freshly resected human white adipose tissue into mature adipocytes (buoyant fraction) and stromal vascular fraction (SVF). ECSCR is prominent on lipid-bearing, buoyant white adipocytes. SVF antigen CD34 is detected only in the pelleted fraction. **D.** Confocal micrographs of human muscle and WAT tissue sections stained with UEA-1 lectin (Red) to show vasculature and, anti-ECSCR antiserum (Green). Top: Skeletal muscle. ECSCR is present on the capillaries and on an unidentified resident cell type. Striated muscle fibers are negative for ECSCR. Bottom: White adipose tissue. Adipocyte profiles, immune reactive for ECSCR but negative for UEA lectin, are indicated with (*).

### Blood collection

After 6 h of fasting, approximately 1% (W/V) of mice body weight blood was withdrawn under anesthesia via submandibular vein puncture. For plasma measurements of the fed state, blood withdrawal was performed in the morning prior to 8 AM, i.e. within one hour of the regular lights on cycle for the colony. Serum was separated by centrifugation at 1000g for 10 min at 4°C and either stored in -20°C or immediately used for analysis.

### Isolation of stromal cell fraction and *in vitro* adipocyte differentiation

Male 8–12 week old mice were euthanized and. epididymal fat pads excised immediately under aseptic conditions. Stromal vascular cell fractions were isolated and cultured in vitro as described previously [14]. Briefly, fat pads were minced into small pieces using sterile fine tip scissors. The resultant tissue suspension was digested with 0.1% collagenase in DMEM-F12 medium for 1hr at 37°C, and cells were pelleted by centrifugation at 400g for 10 min. Red blood cell contamination was removed by suspending the pellet in RBC lysis buffer (150 mM NH_4_Cl, 10 mM, 0.1 mMEDTA and KHCO_3_). The treated suspension was then passed through a 40 μm cell strainer to remove endothelial cell contamination. DMEM-F12 medium with 20% FBS was then added (1:1) to the cell suspension, and the stromal cell fraction was pelleted a second time by centrifugation at 400g for 10 min. The isolated cell pellet was re-suspended in culture maintenance medium (DMEM-F12 medium with 10% FBS and 1% antibiotic and anti-mycotic mixture) and plated on tissue culture plastic. After overnight culture, non- adherent cells were removed by aspiration. The resulting stromal vascular cell preparation was then used for adipocyte differentiation. Adipogenic differentiation was induced by incubating the confluent cell monolayers with fresh DMEM:F12 medium with 10% FBS containing 0.5 μM 1-methyl-3 isobutylxanthine, 1μM dexamethasone, 10 μg/ml insulin and 100 μM indomethacin for 48 hr, and then cultures in DMEM:F12 medium with 10% FBS containing 10 μg/ml insulin for 3 weeks.

### Human adipose tissue samples

Research on human samples was approved by The Medical College of Wisconsin Institutional Review Board #5 (Protocol PRO00018298) and written informed consent was obtained in accordance with the Declaration of Helsinki. Human adipose tissue samples were obtained as discarded tissue resected from otherwise healthy patients undergoing liposuction. Adipose blocks were embedded in paraffin and sectioned following standard procedures. Additional paraffin sections of human tissues were obtained from US Biomax.

### Cell culture and transfections

3T3-L1 cells were maintained on tissue culture plastic unless indicated otherwise. 3T3-L1 cells were purchased from ATCC and cultured in DMEM supplemented with 10% bovine serum with antibiotic and anti-mycotic mixture for four to ten passages. Differentiation of 3T3-L1 pre-adipocytes to mature adipocytes was achieved as described previously [15]. For knockdown of *Ecscr*, 3T3-L1 cells were infected with lentiviral particles constituting ECSCR targeting shRNA sequence in pLKO.1 vector(Addgene). After 24 h, transduced cells were selected with 2 μg/ml puromycin. For controls, cell infected with lentiviral particles carrying non-targeting scrambled shRNA in pLKO.1 vector. Puromycin resistant polyclonal cultures were differentiated into matured adipocytes as described above. Stromal vascular cell fraction of WAT was isolated, cultured and differentiated into matured adipocytes as described previously [14]. 293T cells were cultured in DMEM with 10% FBS in plastic tissue culture dishes. Transfections of 293T cells were performed using Lipofectamine 2000 (ThermoFisher) following the manufacturer’s instructions.

### Cell treatments

Differentiated cells were serum starved overnight in phenol red free DMEM then stimulated with either 10 μg/ml insulin or 10 μM isoproterenol for 15 min. Cell lysates were prepared in RIPA buffer containing protease and phosphatase inhibitors after washing three times with ice-cold PBS, except for co-immunoprecipitation (co-IP) experiments. For co-IP experiments, cells were suspended in 10 mM HEPES buffer containing 0.1% Triton X100 plus protease and phosphatase inhibitors. Cell lysates were prepared by repeated passage (20 strokes) of cell suspensions through a 27 gauge needle. The lysates were then clarified by centrifugation at 12000g for 15 min. Protein content measured with a Bio-Rad Dc protein assay kit (Cat#5000111).

### Western blotting and densitometry

Thirty μg/ of cell lysates were resolved on 4–20% gradient SDS-PAGE and then transferred to PVDF membranes. Membranes were blocked with 5%BSA in Tris buffered saline (pH 8.0) with 0.1% Tween 20 then probed overnight at 4°C with primary antisera(1:1000 dilution) as indicated in Fig legends. The blots were then probed using horseradish peroxidase (HRP)-conjugated secondary antibodies, and immune reactive protein bands were visualized using chemi-luminescence substrate photoreactive X-ray films. Densitometry quantification of protein band intensity was performed using NIH image-J software.

### Measurement of triglyceride content in lipoproteins

Serum esterified TGs and free glycerol content were measured using a triglyceride assay kit (Sigma; Cat#FG0100-1KT) following the manufacturer’s protocol. Triglyceride content of HDL and LDL/VLDL fractions was determined using the precipitating reagent method (Cell Biolabs Inc; Cat# 2369054). Briefly, LDL/VLDL precipitating reagent was mixed 1:1 with serum, and the LDL/VLDL fraction was separated by centrifugation at 2000 g for 15 min. Triglyceride content in the supernatant (HDL fraction) and precipitate (LDL/VLDL fraction) was then assayed as glycerol as described above.

### Measurement of free glycerol in culture media

Differentiated *ex vivo* adipocytes were washed and serum starved overnight in phenol red free DMEM. One mililiter of medium was collected from each well, heated to 75°C for 15 min to inactivate lipases and then free glycerol content assayed using the free glycerol reagent (Sigma) following the manufacturer’s protocol. Glycerol levels were normalized to the incubation period and to the protein content in the respective wells.

## Results

### ECSCR protein in white adipose tissue (WAT) stromal and mature adipocytes

In a survey blot of ECSCR protein in mouse tissues using anti-ECSCR antiserum[8], high levels were detected in WAT tissue lysates (**Fig 1A**), but not brown adipose tissue (data not shown). Since the ECSCR protein signal in WAT appeared to exceed that in other highly vascularized tissues such as liver or kidney, we hypothesized that the protein signal in this tissue reflected non-vascular expression. To investigate ECSCR expression in adipocytes, we used the 3T3-L1 adipocyte differentiation model (**Fig 1B**). During the course of differentiation of 3T3-L1 toward a lipid-storing phenotype, we found a steady increase in ECSCR protein levels, with approximately 3-fold greater normalized ECSCR expression (average 3.2 ± 0.7 fold increase by densitometry in 3 independent experiments, p<0.05, Student’s T-test) in the differentiated cells compared to the undifferentiated parental population. ECSCR protein increase was less pronounced than that of the lipid droplet associated protein perilipin over the same differentiation course[16].

To further clarify cell types within WAT that express ECSCR, we fractionated human adipose tissue into the buoyant mature adipocytes and the denser Stromal Vascular Fraction (SVF) containing endothelial cells, adipocyte precursors, and other cells (**Fig 1C**). ECSCR protein was detected in both fractions, but highly enriched in the buoyant fraction. Finally, we used anti-ECSCR immunohistochemistry and confocal microscopy on human tissue sections (**Fig 1D**), using UEA-1 lectin to counterstain for microvasculature. In muscle, ECSCR is highly expressed on the lectin positive microvasculature. In WAT, ECSCR is present on vessels, to a lesser extent on the perivascular cells, and on enlarged cells histologically resembling white adipocytes. Taking the mouse tissue, human fractionation and staining data together, we conclude that ECSCR is expressed on white stromal and mature adipocytes in addition to endothelial cells.

### Elevated circulating triglycerides and free fatty acids in ECSCR null mice

To investigate *Ecscr* gene function *in vivo*, we generated and validated *Ecscr* knockout mice using ES cells generated by the Knockout Mouse Project (KOMP) [17] repository (**Panel A in S1 Fig**). Mice lacking *Ecscr* were homozygous viable and fertile and recovered in the expected Mendelian frequency, as has been reported elsewhere [18]. Lung lysates of mice homozygous for our *Ecscr* targeted allele showed no detectable ECSCR protein (**Panel B in S1 Fig**). Although loss of *Ecscr* reduces VEGFR2 signaling [8, 9], we did not observe major angiogenesis defects in *Ecscr* ^*-/-*^ mice (data not shown).

**Fig 2:**
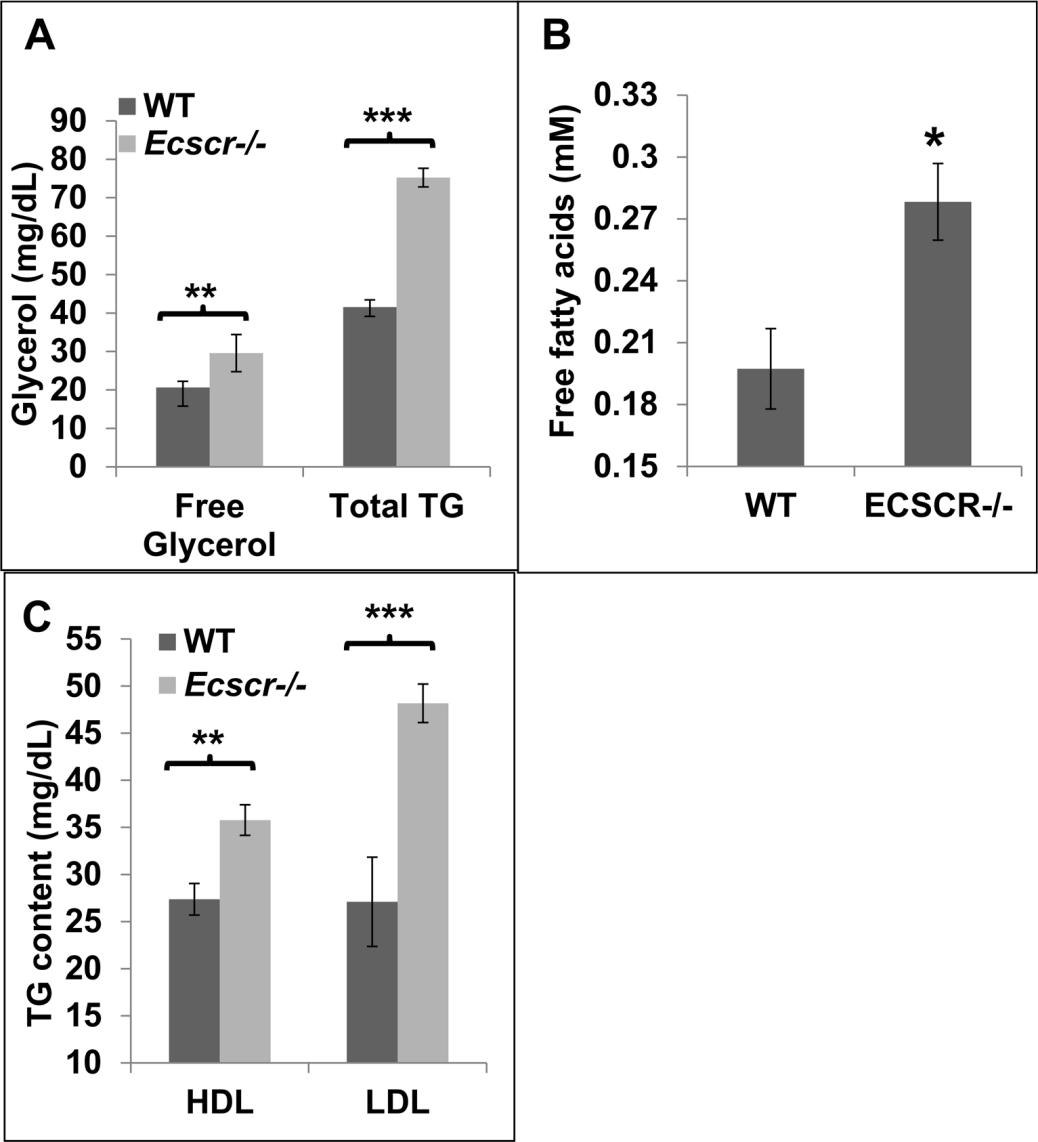
Elevated serum triglycerides and free fatty acids in fasting *Ecscr* ^-/-^ mice. Mice were fasted and plasma lipids analyzed as indicated. Parameters are plotted as mean ± SE (n≥5) for all quantities measured. **A.** Free fatty acid determination (as glycerol, left) and total triglycerides (TGs, right). Both esterified TGs and free fatty acid are elevated in fasting ***Ecscr*** ^**-/-**^ mice. **B.** Direct determination of free fatty acids **C**. TG partitioning in fractionation of circulating lipids into HDL supernatant and mixed lipoprotein pellet. Elevation of TGs in *Ecscr* ^*-/-*^ mice is associated chiefly with the mixed lipoprotein supernatant fraction. For all panels, *, p<0.05, **, p<0.01, ***, p<0.001, Student’s T-test.

Prompted by ECSCR expression on lipid-bearing adipocytes, we compared circulating serum lipids in wild-type (WT) and knockout (*Ecscr* ^*-/-*^) mice (**Fig 2**). Fasting *Ecscr* ^*-/-*^ mice showed significantly elevated serum free fatty acids (FFA; measured as free glycerol), and also esterified triglycerides (TGs; **Fig 2A**). We confirmed the presence of elevated FFAs via direct fatty acid detection (**Fig 2B**). The excess FFA and TG were associated with both high density and low-density lipoprotein fractions (**Fig 2C**). We also investigated WAT morphology in *Ecscr* ^*-/-*^ mice (**S2 Fig)**. Quantification of adipocyte profile areas in epididymal WAT of adult male mice (**Panel C in S2 Fig**) and weight gain studies (**Panel D. in S2 Fig**) did not reveal major differences between WT and *Ecscr* ^*-/-*^ mice, although the modal adipocyte area size was somewhat reduced (data not shown). Total plasma cholesterol and HDL-associated plasma cholesterol were not different in *Ecscr* ^*-/-*^ mice (**Panel E in S2 Fig**). These results are consistent with a modest basal lipolysis increase in fasting *Ecscr* ^*-/-*^ mice.

### PI3K/AKT and insulin signaling in adipocytes is affected by loss of ECSCR

**Fig 3:**
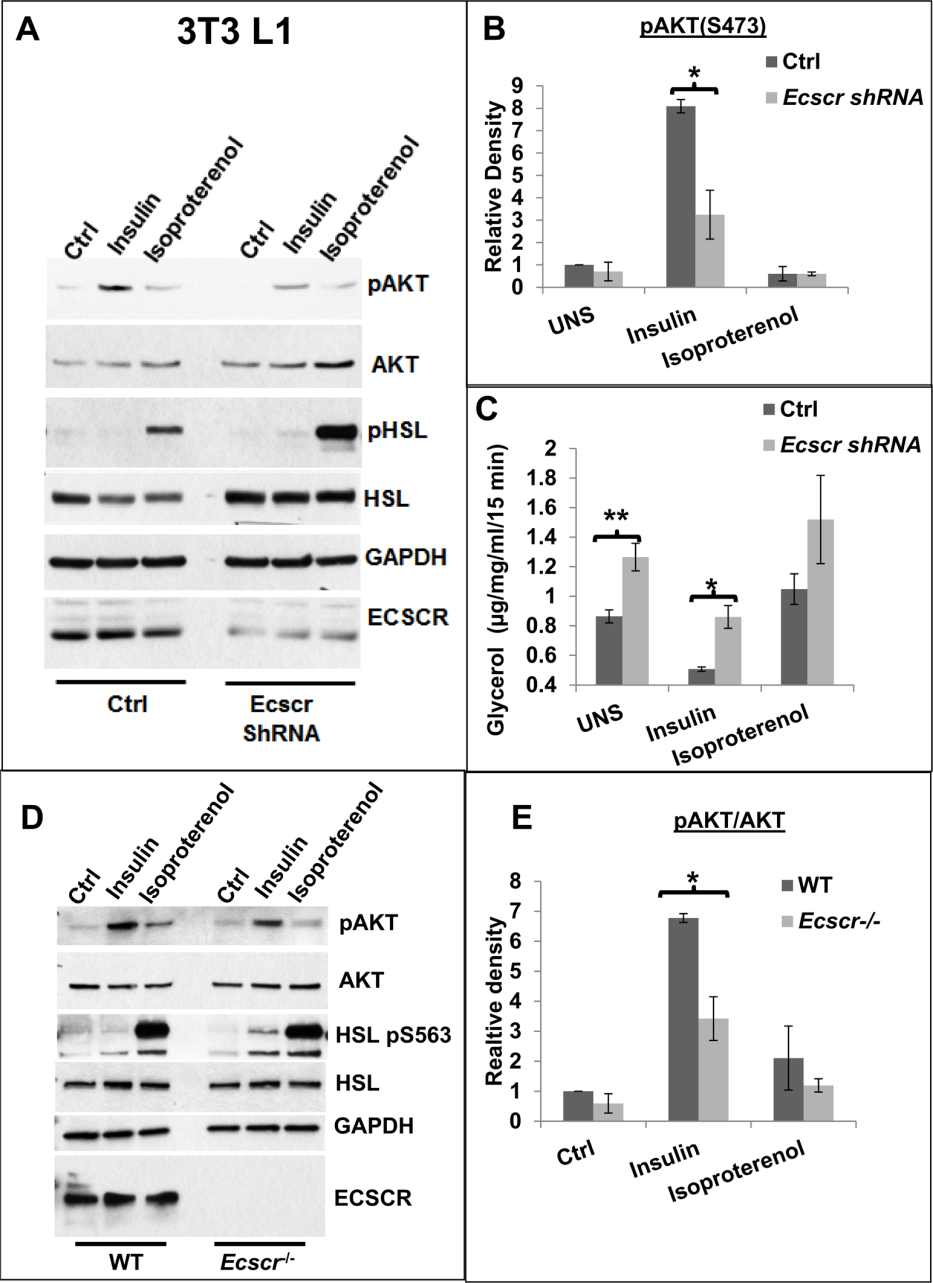
*Ecscr* silenced 3T3-L1 adipocytes and *ex vivo* differentiated *Ecscr* ^-/-^ adipocyte cells show reduced Akt phosphorylation. **A-C.** 3T3-L1 pre-adipocytes were transduced with lentiviral control shRNA or *Ecscr*-targeting shRNA and allowed to differentiate after puromycin selection. Adipocytes were then stimulated with insulin or isoproterenol and analyzed for lipolysis pathways (**A and B**), and glycerol release (**C**). Results are presented as mean +/- SEM of three independent experiments. Similar results were obtained with a second, independent, *Ecscr* targeting shRNA (not shown). **D and E.** Stromal vascular fraction cells obtained from wild-type (left) or *Ecscr* ^-/-^ mice (right) were differentiated into mature adipocytes, stimulated as indicated, then analyzed for lipolysis relevant phospho-epitopes. **D.** Ex-vivo adipocytes from *Ecscr* ^-/-^mice show deficient insulin-dependent AKT activation. **E.** Densitometry quantitation of pAKT (s473) blots (mean +/- SEM of three independent experiments. For panels **B,C** and E, *, p<0.05, **, p<0.01, Student’s T-test.

We next examined the effect of *Ecscr* deficiency *in vitro* using 3T3-L1 cells. 3T3-L1 cells were transduced with control or *Ecscr* targeting shRNA constructs, differentiated to adipocytes, and stimulated with insulin or with the catecholaminergic stimulus isoproterenol (**Fig 3**). *Ecscr* knockdown cells showed a significantly blunted AKT phosphorylation in response to insulin stimulation (**Fig 3A and 3B**). Glycerol release was also elevated in basal and in insulin-stimulated adipocytes lacking ECSCR (**Fig 3C**). There was also a trend toward increased phosphorylation of the lipolytic enzyme HSL in *Ecscr* silenced differentiated cells (data not shown).

To confirm changes in *Ecscr* deficient cells, we isolated stromal vascular fraction adipocyte precursors from knockout mice, differentiated them to adipocytes, and again stimulated with insulin or isoproterenol (**Fig 3D**). *Ecscr* ^*-/-*^ ex-vivo adipocytes showed significantly reduced insulin-stimulated AKT phosphorylation (**Fig 3D and 3E**) and a trend toward increased HSL phosphorylation (not shown). Finally, similar alterations in phospho-AKT and phospho-HSL were seen in acutely isolated fat pad (**S3 Fig**). Taken together, these findings suggest that basal lipolysis and lipolysis following insulin stimulation are elevated in *Ecscr* ^*-/-*^ mice.

### Insulin signaling increases ECSCR interaction with PTEN

**Fig 4:**
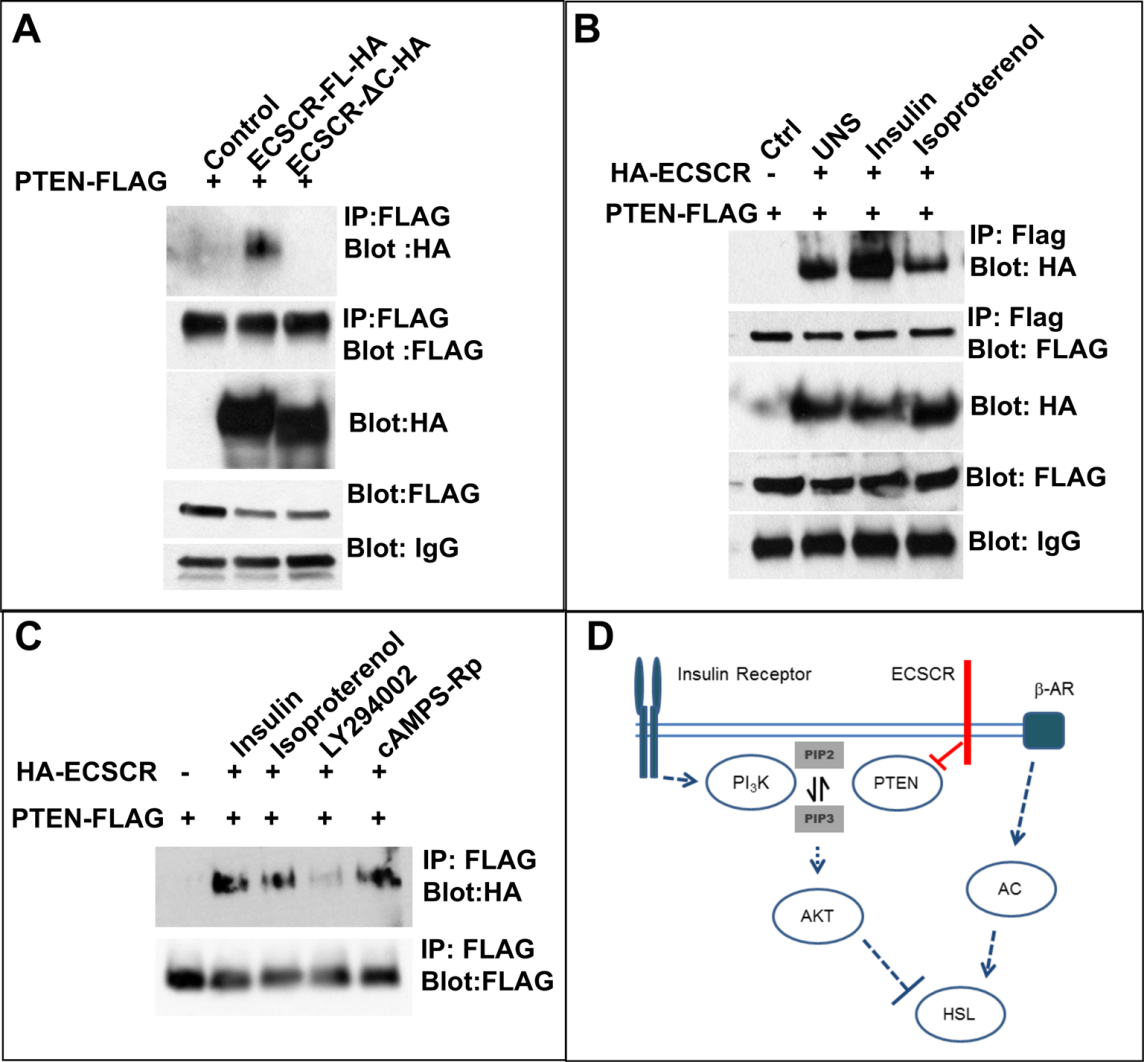
Insulin stimulation increases association between ECSCR C-terminus and PTEN in 293T cell overexpression system. **A.** 293T cells were co-transfected with FLAG-tagged PTEN and either full-length or cytoplasmic truncated ECSCR and subjected to anti-FLAG immune precipitation. Full-length, but not truncated, ECSCR co-immune precipitates with PTEN. **B.** Insulin stimulation increases ECSCR-PTEN association. **C.** PI3K inhibitor LY294002 but not by PKA inhibitor cAMPS-Rp blocks ECSCR-PTEN association. Blots are representative of three independent experiments. **D**. Model for ECSCR influence on insulin inhibition of HSL in white adipocytes. ECSCR interaction with PTEN is predicted to inhibit its activity, allowing increased insulin activation of the PI3K-AKT axis. *Dashed lines* represent indirect interactions.

To investigate how ECSCR could affect the lipolysis pathway, we focused on the reported ECSCR interaction with PTEN [18], which is implicated in control of insulin-dependent AKT phosphorylation [3]. In a 293T cell transfection system (**Fig 4**), full-length ECSCR, but not ECSCR lacking cytoplasmic sequences, could immune precipitate PTEN (**Fig 4A**). Insulin stimulation increased ECSCR-PTEN complex formation, while isoproterenol reduced this complex formation (**Fig 4B**). These results were confirmed with endogenous PTEN and ECSCR in 3T3-L1 pre-adipocyte cells (**S4 Fig**). To investigate intracellular signals important in ECSCR-PTEN complex formation, we focused on PI3K-AKT pathway and protein kinase A (PKA) pathway [19]. The PI3 Kinase inhibitor LY294002 (10 μM), but not the PKA inhibitor cAMPS-Rp (100 μM), blocked the formation of the ECSCR-PTEN complex following insulin stimulation (**Fig 4C**). Taken together, these results suggest that insulin-mediated signals in adipocytes are influenced by ECSCR, and ECSCR-PTEN complex formation is sensitive to insulin and may contribute to ECSCR lipolysis modulation in adipocytes.

## Discussion

This study reports the role of ECSCR protein in modulating lipolysis via its impact on insulin-mediated pAKT signaling in adipocytes. The salient features of this study include the characterization of ECSCR protein expression in mouse and human WAT tissue and cells, the identification of ECSCR as a lipolysis regulator in mammals, the influence of ECSCR on insulin signaling in adipocytes, and finally the potential mechanistic role of ECSCR-PTEN interaction in enhancing insulin-PI3K-AKT suppression of lipolysis.

### *Ecscr* messenger RNA levels do not predict protein levels

ECSCR protein is expressed on mature and maturing white adipocytes both *in vivo* and in 3T3-L1 cells as they mature to adipocytes. This is in contrast to *Ecscr* messenger RNA, which is not highly enriched in WAT nor in differentiating 3T3-L1 cells [11] (data not shown). This disparity between messenger RNA and protein results raises the possibility that the protein stability of ECSCR is tightly regulated. This control of protein stability might further explain the consistent elevation of PTEN protein observed in white adipose tissue in *Ecscr* ^*-/-*^ mice (S4 Fig and data not shown). Indeed, these results are consistent with our previous results [8] that suggested that ECSCR protein influences cell trafficking, with impacts on VEGFR-2 protein stability and cellular signaling.

### Cells lacking *Ecscr* show reduced Akt phosphorylation

Elevated circulating triglycerides in *Ecscr* ^*-/-*^ mice were reproduced *in vitro* in the 3T3-L1 adipocyte system (**Fig 3C**). Cultured 3T3-L1 adipocytes silenced for *Ecscr*, and *ex vivo* adipocytes from *Ecscr* ^*-/-*^ mice, showed reduced basal and insulin-stimulated AKT activation and excess lipid release. Mechanistically, increased basal lipolysis observed in *Ecscr* silenced 3T3-L1 cells could result from increased PTEN activity, which would reduce AKT activation leading to elevated HSL phosphorylation. Our overexpression studies results further suggest that insulin stimulation triggers PI3K-dependent formation of an ECSCR-PTEN complex. This complex could serve to amplify PI3K dependent signals, perhaps via physical removal of PTEN from sites of signaling lipid generation [20].

*Ecscr* ^*-/-*^ mice reported elsewhere [2, 16] have consistently shown alterations in the PI3K-AKT axis. Akakabe et al.[11] have studied insulin responses of *Ecscr* ^*-/-*^ mice in detail, and have shown that *Ecscr* ^*-/-*^ mice have improved glucose clearance, increased systemic insulin sensitivity and increased endothelial AKT phosphorylation in response to insulin. Our results in white adipocytes, conversely, indicated that loss of *Ecscr* in this cell type reduces insulin-AKT signaling. In particular, we show a reduction in insulin-dependent changes in AKT and HSL phosphorylation, and reduced suppression of lipolysis in insulin stimulated cultured adipocytes lacking *Ecscr* (**Fig 3**). Biochemical differences observed in *Ecscr* deficient cells might arise due to different concentrations of insulin used by the two labs to stimulate cells. Our laboratory stimulates adipocytes at the LD50 for the insulin receptor, 2 nM, corresponding to the half-maximal binding of insulin receptors in adipocytes [21]. The Akakabe group [11] reported using insulin at saturating levels of 100 nM. The differing results suggest that ECSCR influences on AKT activation are sensitive to insulin concentrations. A second possible explanation for differential ECSCR impact on insulin-PI3/AKT activation would be the different cell type studied in culture and in whole-animal measures. Glucose clearance from the blood is dominated by skeletal muscle [22], and in this tissue ECSCR is primarily restricted to the microvasculature (**Fig 1D**). Conversely, lipid storage is dominated by WAT, and in that tissue ECSCR is detected on vessels and adipocytes. These results may therefore have relevance for local tissue insulin sensitivity [23–25]. Further studies including conditional deletion of the *Ecscr* gene from white adipocytes and from endothelial cells could resolve the contributions of these two cell types to the reported metabolic phenotypes. One scenario encompassing both sets of data would be that endothelial specific *Ecscr* deleted mice would have normal altered glucose tolerance, while white adipocyte specific deletion would result in mice with elevated plasma triglycerides.

The prominent plasma lipid elevation in fasting *Ecscr* ^*-/-*^ mice and blunted insulin response in *Ecscr* silenced cells suggested impairment in insulin-dependent energy storage in white adipocytes. However, *Ecscr* deficient animals only showed modest alterations in adipose tissue histology (**S2 Fig**) and no significant difference in initial weight gain in response to high-fat diet(**Panel D in S2 Fig**). Final resolution of this paradox would require kinetic study of white adipocyte storage and release and studies of the lipid species composition within white adipocytes lacking *Ecscr* [26, 27]. Decreased insulin control of adipocyte lipid storage is an important initiating event leading to metabolic syndrome and associated morbidities. ECSCR function seems to describe a control point for fat storage, and thus ECSCR could become a drug target for metabolic syndrome. These and future studies will refine this hypothesis further.

## Supporting Information

S1 Fig Generation and validation of *Ecscr* null allele A. *Ecscr* “Knockout first” targeting allele generated and transfected into EC cells by the KOMP project (schematic adapted from their web page; see Methods for exact project number). The knockout first allele inserts neomycin resistance and **β-**galactosidase cassettes downstream of exon 2. **B.** Western blot analysis of ECSCR protein levels in adult mouse lung of the indicated genotypes. No protein is detected in animals homozygous for the *Ecscr* knockout first allele.
(TIF)

S2 Fig Analysis of WAT histology and plasma cholesterol in *Ecscr* ^-/-^ mice. **A, B.** Hematoxylin/Eosin stain of representative sections of epididymal WAT from wild-type (**A**) or *Ecscr* ^-/-^ (**B**) adult males. Adipose tissue in *Ecscr* ^-/-^ mice has grossly normal appearance. **C.** Histogram of WAT profile areas observed in epididymal WAT of adult male wild-type (black bars) and *Ecscr* ^-/-^ (white bars) mice. **D.** Cohorts of 3 female mice of the indicated genotypes were fed a HFD (50% of calories from fat; Research Diets D12492) beginning at weaning and weighed weekly. **E**. Serum cholesterol levels in fasted wild-type or *Ecscr* ^-/-^ mice. Neither total cholesterol nor HDL associated cholesterol levels are significantly different between wild-type and *Ecscr* ^-/-^ mice. Results are presented as the mean +/- SEM for at least 3 different animals.
(TIF)

S3 Fig WAT of mice lacking *Ecscr* shows depressed AKT activation, enhanced HSL phosphorylation, and elevated PTEN protein. Western blot analysis (**A**) and densitometry quantification (**B**-**D**) of lipolysis related proteins in epididymal WAT obtained from adult male wild-type and *Ecscr* ^*-/-*^ mice. Each vertical lane represents tissue lysate obtained from a different mouse of the indicated genotype.
(TIF)

S4 Fig Elevated endogenous phospho-AKT in 293T cells overexpressing ECSCR. A. 293T cells were transiently transfected with control vector or HA- tagged ECSCR and analyzed by western blot. Phospho-Akt levels are consistently elevated in overexpressing cells in this system. B. Normalized pAKT/AKT densitometry in 293T cells overexpressing ECSCR. Results are presented as mean +/- SEM for 3 independent experiments. *, p<0.05, Student’s 1-tailed T test.
(TIF)

S5 Fig Regulated endogenous ECSCR-PTEN Interactions in differentiated 3T3 L1 cells. 3T3-L1 cells were differentiated for 8 days as described in Methods, lysed, and prepared for immune precipitation. Endogenous ECSCR co-immune precipitates with endogenous PTEN, and this interaction is increased following insulin stimulation. Results are representative of three independent experiments.
(TIF)
