# Supplementary figures and images for: Endothelial Cell Surface Expressed Chemotaxis and Apoptosis Regulator (ECSCR) Regulates Lipolysis in White Adipocytes via the PTEN/AKT Signaling Pathway
Source: PLoS One. 2015 Dec 21;10(12):e0144185. doi: 10.1371/journal.pone.0144185 (PMC4686900; doi:10.1371/journal.pone.0144185)

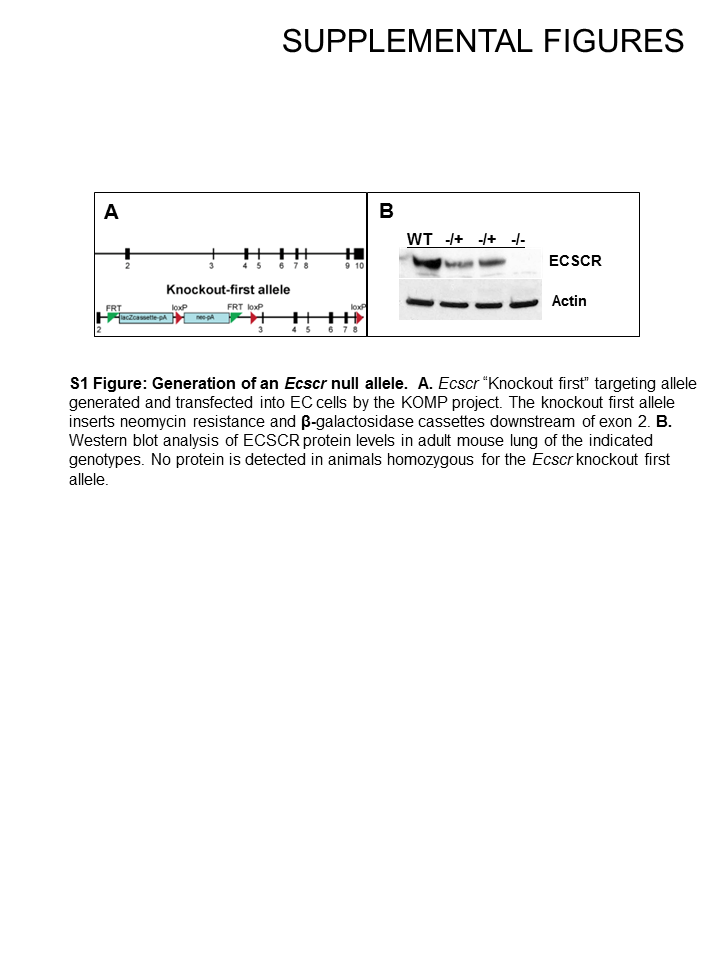

Supplement: S1 Fig — Ecscr “Knockout first” targeting allele generated and transfected into EC cells by the KOMP project (schematic adapted from their web page; see Methods for exact project number). The knockout first allele inserts neomycin resistance and β-galactosidase cassettes downstream of exon 2. B. Western blot analysis of ECSCR protein levels in adult mouse lung of the indicated genotypes. No protein is detected in animals homozygous for the Ecscr knockout first allele. (TIF) [file pone.0144185.s001.TIF]

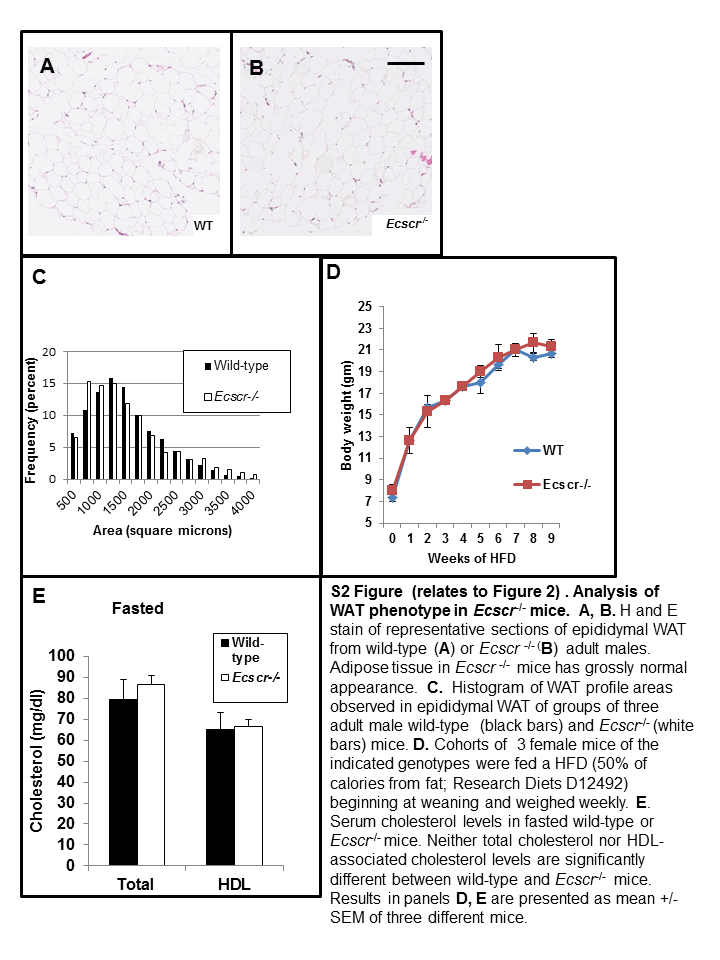

Supplement: S2 Fig — A, B. Hematoxylin/Eosin stain of representative sections of epididymal WAT from wild-type (A) or Ecscr -/- (B) adult males. Adipose tissue in Ecscr -/- mice has grossly normal appearance. C. Histogram of WAT profile areas observed in epididymal WAT of adult male wild-type (black bars) and Ecscr -/- (white bars) mice. D. Cohorts of 3 female mice of the indicated genotypes were fed a HFD (50% of calories from fat; Research Diets D12492) beginning at weaning and weighed weekly. E. Serum cholesterol levels in fasted wild-type or Ecscr -/- mice. Neither total cholesterol nor HDL associated cholesterol levels are significantly different between wild-type and Ecscr -/- mice. Results are presented as the mean +/- SEM for at least 3 different animals. (TIF) [file pone.0144185.s002.TIF]

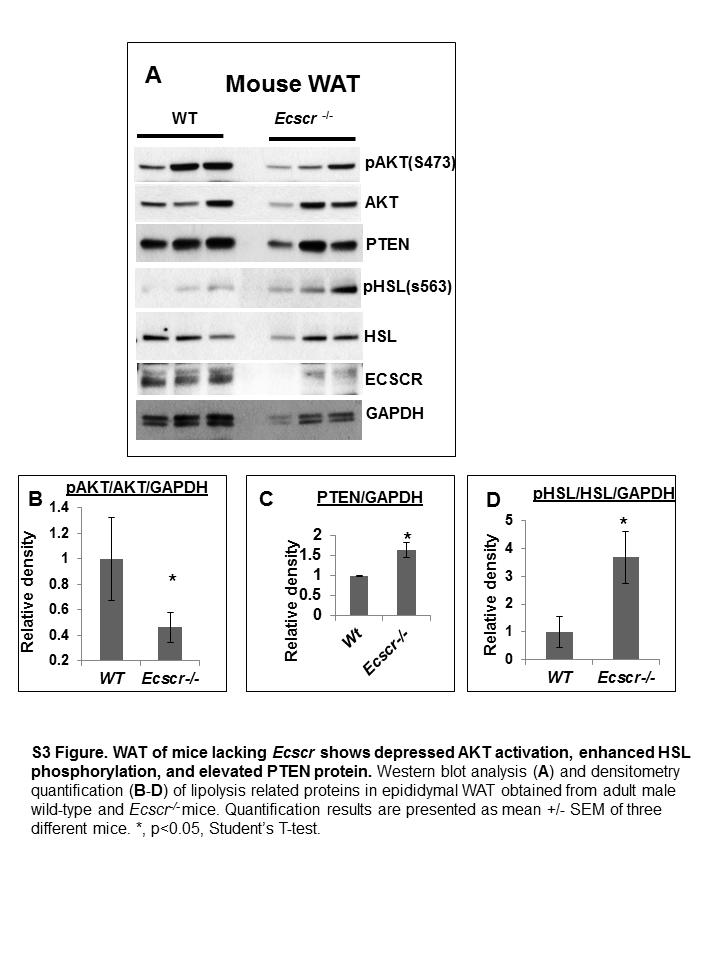

Supplement: S3 Fig — Western blot analysis (A) and densitometry quantification (B-D) of lipolysis related proteins in epididymal WAT obtained from adult male wild-type and Ecscr -/- mice. Each vertical lane represents tissue lysate obtained from a different mouse of the indicated genotype. (TIF) [file pone.0144185.s003.TIF]

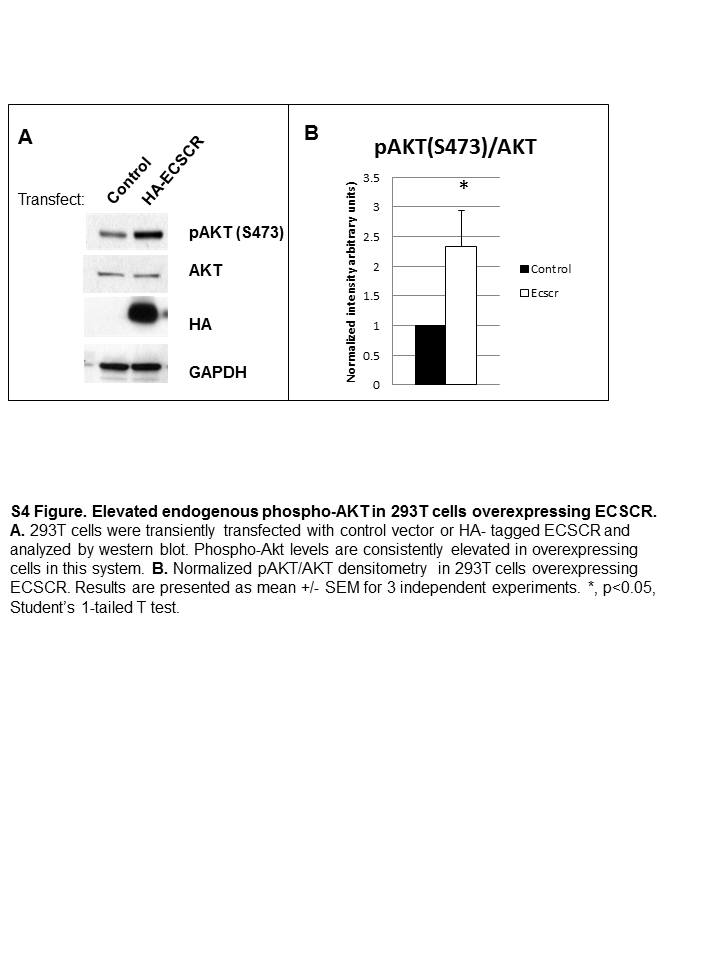

Supplement: S4 Fig — A. 293T cells were transiently transfected with control vector or HA- tagged ECSCR and analyzed by western blot. Phospho-Akt levels are consistently elevated in overexpressing cells in this system. B. Normalized pAKT/AKT densitometry in 293T cells overexpressing ECSCR. Results are presented as mean +/- SEM for 3 independent experiments. *, p<0.05, Student’s 1-tailed T test. (TIF) [file pone.0144185.s004.TIF]

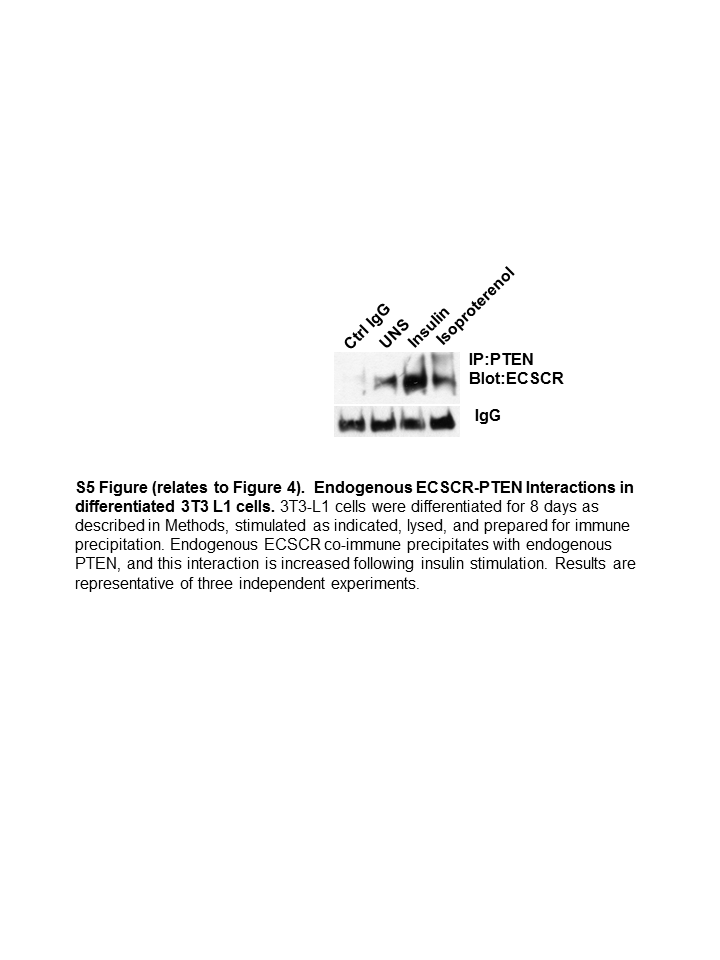

Supplement: S5 Fig — 3T3-L1 cells were differentiated for 8 days as described in Methods, lysed, and prepared for immune precipitation. Endogenous ECSCR co-immune precipitates with endogenous PTEN, and this interaction is increased following insulin stimulation. Results are representative of three independent experiments. (TIF) [file pone.0144185.s005.TIF]
